# Sex differences in adults with acute myeloid leukemia and the impact of sex on overall survival

**DOI:** 10.1002/cam4.5461

**Published:** 2022-11-23

**Authors:** Nickolas Stabellini, Benjamin Tomlinson, Jennifer Cullen, John Shanahan, Kristin Waite, Alberto J. Montero, Jill S. Barnholtz‐Sloan, Nelson Hamerschlak

**Affiliations:** ^1^ Graduate Education Office Case Western Reserve University School of Medicine Cleveland Ohio USA; ^2^ Department of Hematology‐Oncology University Hospitals Seidman Cancer Center Cleveland Ohio USA; ^3^ Faculdade Israelita de Ciências da Saúde Albert Einstein Hospital Israelita Albert Einstein São Paulo Brazil; ^4^ Department of Population and Quantitative Health Sciences Case Western Reserve University School of Medicine Cleveland Ohio USA; ^5^ Case Comprehensive Cancer Center Cleveland Ohio USA; ^6^ Cancer Informatics Seidman Cancer Center at University Hospitals of Cleveland Cleveland Ohio USA; ^7^ Division of Cancer Epidemiology and Genetics (DCEG), Trans‐Divisional Research Program (TDRP) National Cancer Institute, National Institutes of Health Bethesda Maryland USA; ^8^ Center for Biomedical Informatics and Information Technology (CBIIT) National Cancer Institute, National Institutes of Health Bethesda Maryland USA; ^9^ Oncohematology Department Hospital Israelita Albert Einstein São Paulo Brazil

**Keywords:** acute myeloid leukemia, AML, sex differences, survival, treatment, treatment adverse events

## Abstract

**Background:**

There is a male predominance of acute myeloid leukemia (AML) incidence, but survival data are conflicting. The objective of this study is to carry out a comprehensive analysis of sex differences in AML, and to investigate the impact of sex disparities in survival.

**Methods:**

The cohort included patients ≥18 years diagnosed with AML (2010–2022). Demographics, treatment patterns, treatment adverse events, and survival were analyzed. The population was described and compared by sex, and sex‐based risks and associations were obtained via Cox proportional‐hazards regression.

**Results:**

In total, 1020 AML patients were analyzed (57.4% males), with lower risk of death for females (aHR = 0.41, 95% CI 0.26–0.66). Among females, BMT (aHR = 0.51, 95% CI 0.27–0.97), hospitalization record (aHR = 0.65, 95%CI 0.45–0.93), and higher appointment completion rates (aHR = 0.98, 95% CI 0.98–0.98) were associated with lower risk of death. Overall, and similarly in males, higher age at diagnosis (aHR = 1.03, 95% CI 1.02–1.04) and a TP53 mutation (aHR = 2.24, 95% CI 1.69–2.97) were associated with higher risk of death.

**Conclusion:**

Sex differences exist in both AML incidence and overall survival. Treatment and health care factors should be addressed by caregivers and public policies developed to reduce mortality rates and mitigate existing sex differences.

## Graphical abstract

This study, focused on sex differences in acute myeloid leukemia, reported a 1.34 male:female ratio with a later start of treatment and lower treatment adherence rates in females. Bone marrow transplant, hospitalization, and higher treatment completion rates were associated with lower risk of death in females. In patients with a hospitalization record, females were 39% less likely to receive chemotherapy, 160% more likely to have a chemotherapy adverse event, and had a 59% lower risk of death.

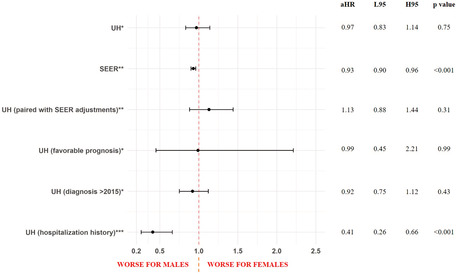

## 1 INTRODUCTION

Cancer is one of the leading causes of death in the world.^1^ Globally, in 2020, it was responsible for 19.3 million cases and 10 million deaths, with expectations that in 2022 it will cause 1,918,030 new cases (~5250 cases per day) and 609,360 deaths (~1700 deaths per day) in the United States (US).^1^, ^2^ Hematologic malignancies (HMs) are one of the cancer types, corresponding, globally, to 6.5% of all cancer cases, with this number rising to 9% in the United States and Europe. Usually, the more prevalent HMs are divided in four subtypes: leukemias (divided into myeloid and lymphoid), Hodgkin lymphomas (HL), non‐Hodgkin lymphomas (NHL), and multiple myelomas (MM).^3^, ^4^

Leukemias, the most distinct group of HMs, comprised 474,519 new cases and 311,594 deaths in 2020 worldwide, with recent declines in the incidence rates of acute lymphocytic leukemia (ALL) and chronic myeloid leukemia (CML) and increases in the rates of chronic lymphocytic leukemia (CLL) and acute myeloid leukemia (AML).^1^, ^4^ AML is the most common acute leukemia in adults and accounts for about a third of all diagnosed leukemias, with its incidence increasing with age.^5^, ^6^ Despite being a very heterogeneous disease, it is categorized into three prognostic risk groups: favorable, intermediate, and adverse.^5^ The treatment is based on induction therapy, seeking to achieve total remission, followed by post‐remission therapy, involving chemotherapy and bone marrow transplantation.^5^

Sex differences have been shown to play an important role in cancer, with incidence rates up to 20% higher and mortality rates up to 40% higher in males.^7^ These differences, although still poorly studied, are also reported in HMs: reports demonstrate a consensus on the male predominance of AML incidence, which increases with age, with data from a recent pan cancer analysis suggesting an overall incidence rate about 1.4 times higher for males.^8^, ^9^, ^10^ On the other hand, survival data are conflicting, with some studies demonstrating better survival and prognosis for females, while others do not demonstrate differences between the sexes.^8^, ^11^, ^12^, ^13^

In addition to conflicting data, the biological underpinnings and specific individual level factors for the reported sex differences in AML are still not fully understood. To our knowledge, there are no studies that establish and clarify the weight of several variables with different characteristics in the differences between sexes in AML. Therefore, the objective of this study is to carry out a comprehensive analysis of sex differences in AML to identify important individual level factors and how they associate with sex differences in overall survival.

## 2 METHODS

### 2.1 Data source

The study setting was the University Hospitals (UH) Seidman Cancer Center. All patient data were obtained from the UH data repository based on the CAISIS platform, which consists of an open‐source, web‐based cancer data management system that integrates eight disparate sources of cancer patient data (i.e., Soarian, NGS Labs, Sunrise Clinical Manager, Tumor Registry, Via Oncology, OnCore, MosiaQ, PRO tools, and others).^14^, ^15^, ^16^ All patient records were de‐identified, and the study was approved by the University Hospitals of Cleveland Institutional Review Board (IRB). All the information obtained from the UH database was subsequently complemented with electronic health record (EHR) information captured via EMERSE (Electronic Medical Record Search Engine) in order to obtain the most accurate and complete information per patient.^17^

The initial cohort included patients ≥18 years diagnosed with acute myeloid leukemia (AML, ICDs 9/10 [International Classification of Diseases] C92.XX and 205.XX, with X standing for any integer) between January 1, 2005, and March 31, 2022. Subsequently, patients were excluded from the analysis if they had a diagnosis of promyelocytic leukemia (ICDs C92.40, C92.41, and C92.42), a diagnosis year before 2010, an unknown diagnosis date, and no sex recorded in the EHR. The study consort diagram is summarized in Figure S1.^18^, ^19^, ^20^

### 2.2 Outcomes

Study outcomes included: (i) treatment and time‐to‐treatment following the cancer diagnosis; (ii) the diagnosis and time‐to‐event of a treatment adverse event; (ii) need for hospitalization and time‐to‐hospitalization following the cancer diagnosis, and (iv) all‐cause overall survival, and time‐to‐death following the cancer diagnosis.

### 2.3 Covariates

The individual level variables analyzed for each eligible patient included: demographics, treatment patterns, treatment adverse events, and overall survival (Figure S2). The demographic characteristics included: age at diagnosis, race (White, Black, other), ethnicity (Hispanic, Non‐Hispanic), smoking status (yes, no, former, unknown), Charlson comorbidity index, prognosis (favorable, other—defined according to molecular and cytogenetic features), and genomic mutations (NPM1, FLT3, MLL, IDH, RUNX1, GATA2, BCR‐ABL1, ASXL1, and TP53).^21^, ^22^

Treatment patterns included appointment completion rate, hospitalizations (including total admissions, elective admissions, non‐elective admissions, and length of stay [LOS]), and the use of a single or combination of treatments during a lifetime: chemotherapy, immunotherapy, and/or bone marrow transplant (BMT). Individual medications analyzed included cytarabine, daunorubicin, idarubicin, midostaurin, sorafenib, gemtuzumab‐ozogamicin, etoposide, fludarabine, clofarabine, mitoxantrone, venetoclax, azacitidine, decitabine, ivosidenib, and enasidenib. Time‐to‐treatment were calculated by subtracting documented first day of therapy from the date of initial diagnosis.

Treatment adverse events were extracted on the basis of ICD, Ninth Revision, and ICD, Tenth Revision, codes, where only diagnoses occurring after the date of first treatment were considered. Complications from any treatment included cognitive decline or dementia (yes, no), and psychological disorders (yes, no) and its subtypes (depression, anxiety, and bipolar disorder).^14^ Complications from immunotherapy were described as immune‐related adverse events (irAEs) (yes, no) and subtypes of irAEs.^14^ Complications from chemotherapy and from BMT were also included. The ICD codes and categorizations are summarized on Table S1.

Overall survival (all cause) information included last follow‐up date, date of death, and vital status (alive or death) after the cancer diagnosis.

### 2.4 Statistical analysis

The study population was described via percentages, median and interquartile range (IQR), and divided into males versus females. The Pearson chi‐square test was used to compare categorical variables. Data distribution assumptions for continuous variables were confirmed using histograms and the Kolmogorov–Smirnov test, followed by the Student's *t*‐tests for normally distributed factors and non‐parametric Kruskal–Wallis tests for non‐normal distributed factors.

The influence of sex on type of treatment received, and treatment adverse events was assessed via hazard ratios (HRs) or adjusted hazard ratios (aHRs) with 95% confidence intervals (CIs) using univariable and multivariable (MV) Cox proportional‐hazards models, after confirming model assumptions, while the influence of sex on hospitalization was assessed via logistic regressions. Overall survival by sex was first assessed using Kaplan–Meier analysis generating median survival by sex with 95% CI and log‐rank tests. Univariable and MV Cox proportional‐hazards regression models were used to predict overall survival by sex after checking assumptions, generating HRs and 95% CI. Subsequently, survival modeling was performed stratified by sex. The influence of the variables on overall survival were obtained from the results of the MV analysis and represented via forest plots.

The covariates selected for MV models were those that achieved a *p* < 0.10 in univariable analyses for the primary outcome and/or those deemed to have clinical importance by study investigators. Independent variable inter‐correlations were examined using correlation plots, and those variables found to be correlated were not included simultaneously in the final multivariable models. A *p*‐value <0.05 was considered significant in the final models, missing values were not included in the final analysis, and males were considered reference for comparison. All analyses were performed using RStudio software.^23^ The STROBE cohort checklist was also used.^24^

### 2.5 Sensitivity analysis and comparison to the general US population

Sensitivity analysis was performed in the following subgroups: patients with a favorable diagnosis, patients diagnosed after 2015—to account for time‐related changes in treatment and practices, and patients with a history of at least one hospitalization. Incidence and mortality findings from our cohort were compared with those from the general US population using the Surveillance, Epidemiology, and End Result (SEER) database.^25^

Data from SEER were obtained from SEER*stat software based on SEER Research Plus database and included patients with AML diagnosis between 2010 and 2019, excluding those with a diagnosis of promyelocytic leukemia. The variables analyzed were categorized after the methodology applied to the UH database and included age at diagnosis, sex, race, ethnicity, chemotherapy, vital status, and median survival.

## 3 RESULTS

Using data from 2010 to 2022, a total of 1020 (22,060 person‐months) AML patients were identified (Table 1). The median age of the cohort was 65 (IQR 53–74) years old, with a predominance of Whites (74.8%) and non‐Hispanics (98.4%). The majority of the patients had a Charlson score ≥ 5 (44.3%), while 9.9% had a favorable prognosis, 39.8% received chemotherapy, 2.6% received immunotherapy, 11.4% undergone a BMT, and 20.8% had at least one hospitalization record during the follow‐up period. Of the patients who received any type of treatment, 33.1% had a psychological affection, and 11.6% had a diagnosis of cognitive decline/dementia. Chemotherapy adverse events were present in 57.2% of those who received this treatment, while irAEs were diagnosed in 5.3% of those treated with immunotherapy, and BMT complications in 74.1% of those who undergone this treatment.

**TABLE 1:** Sex differences in demographics and mutations, acute myeloid leukemia, University Hospitals (UH) population (2010–2022)

	Male	Female	*p* value
	585 (57.4%)	435 (42.6%)	
Age at diagnosis, median (IQR)	66 (54–73)	65 (51–74)	0.48
Race, *n* (%)			
Black	52 (8.9%)	61 (14%)	0.03
White	447 (76.4%)	316 (72.6%)	
Other	86 (14.7%)	58 (13.3%)	
Ethnicity, *n* (%)			
Non‐Hispanic	541 (98.4%)	405 (98.5%)	1
Smoking status, *n* (%)			
Yes	46 (7.9%)	34 (7.8%)	<0.001
No	192 (32.8%)	191 (43.9%)	
Former	220 (37.6%)	107 (24.6%)	
Unknown	127 (21.7%)	103 (23.7%)	
Charlson score, *n* (%)			
1 to 2	116 (19.8%)	98 (22.5%)	0.55
3 to 4	204 (34.9%)	150 (34.5%)	
≥5	265 (45.3%)	187 (43%)	
Favorable prognosis, *n* (%)	62 (10.6%)	39 (9%)	0.44
NPM1 mutation, *n* (%)	22 (3.8%)	22 (5.1%)	0.39
FLT3 mutation, *n* (%)	21 (3.6%)	14 (3.2%)	0.88
MLL mutation, *n* (%)	6 (1%)	2 (0.5%)	0.51
IDH mutation, *n* (%)	29 (5%)	19 (4.4%)	0.77
RUNX1 mutation, *n* (%)	31 (5.3%)	9 (2.1%)	0.01
GATA2 mutation, *n* (%)	4 (0.7%)	3 (0.7%)	1
BCR‐ABL1 mutation, *n* (%)	1 (0.2%)	2 (0.5%)	0.79
ASXL1 mutation, *n* (%)	21 (3.6%)	8 (1.8%)	0.14
TP53 mutation, *n* (%)	32 (5.5%)	33 (7.6%)	0.21

*Note*: A total of 1020 patients were analyzed with 42.6% females. The *p*‐values are result of the Chi‐square test for categorical variables, and the Kruskal‐Wallis test for continuous variables. IQR, interquartile range.

### 3.1 Sex differences in demographics and mutations

Among 1020 patients, 42.6% (*n* = 435) were females, and 57.4% (*n* = 585) were males, establishing a 1.34 male:female ratio. When compared with males, females had higher rates of Black race (14% vs. 8.9%, *p* = 0.03), and never smokers (43.9% vs. 32.8%, *p* < 0.001), and lower rates of RUNX1 mutation (2.1% vs. 5.3%, *p* = 0.01). Sex differences in demographics and mutations are summarized in Table 1.

### 3.2 Sex differences in treatment patterns

**FIGURE 1:**
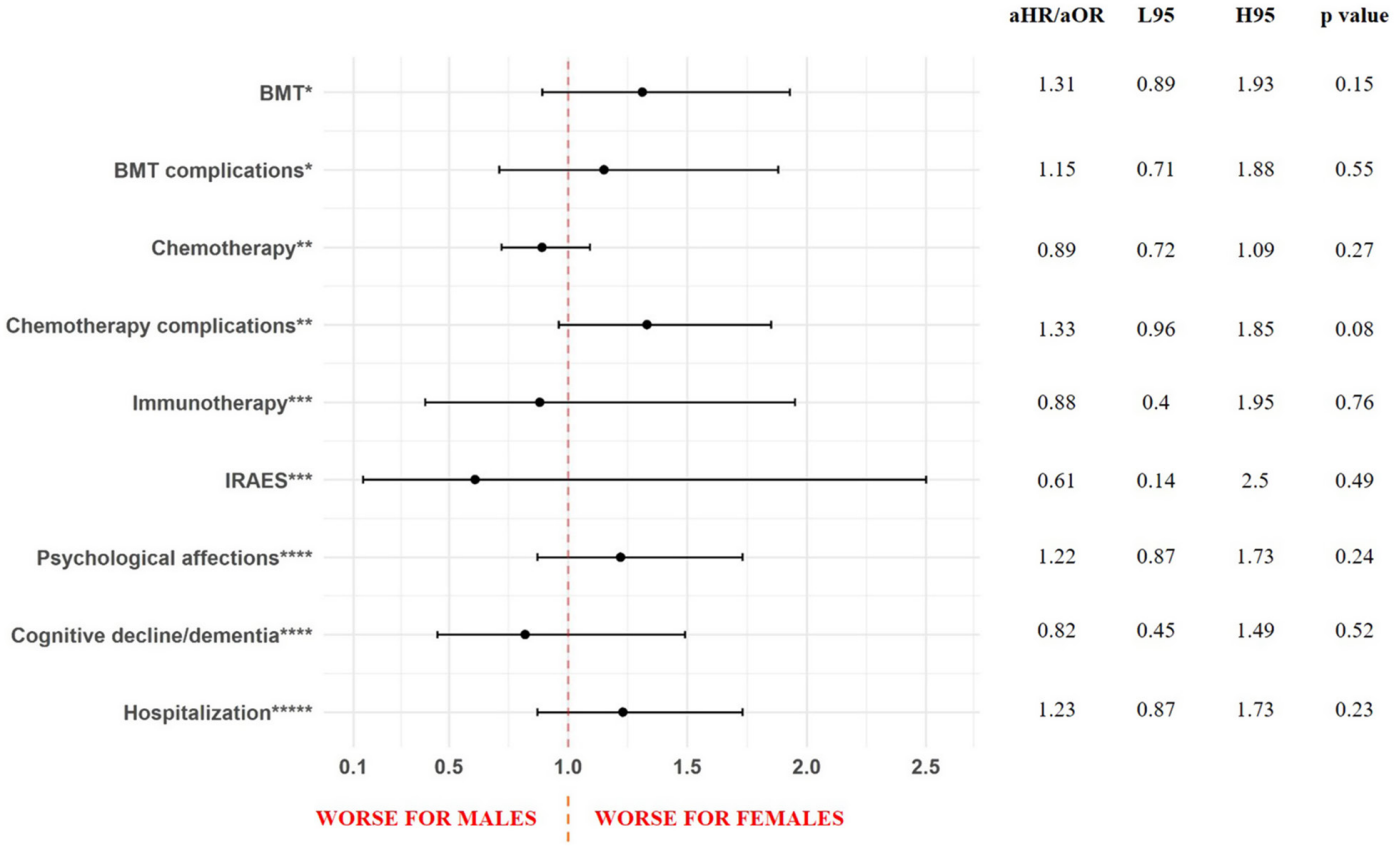
Forest plot representing sex differences in treatment patterns and treatment adverse events, acute myeloid leukemia, University Hospitals (UH) population (2010–2022). Results are presented in adjusted Hazard Ratios (aHR) or adjusted Odds Ratios (aOR) for females, associated with 95% confidence intervals (L95 = lower 95% confidence interval; H95 = higher 95% confidence interval). BMT, bone marrow transplant; IRAES, Immune‐Related Adverse Events. *Adjusted for age at diagnosis, race, ethnicity, Charlson, FLT3 mutation, and prognosis. **Adjusted for age at diagnosis, race, Charlson, and prognosis. ***Adjusted for race, FLT3, RUNX1. ****Adjusted for age at diagnosis, Charlson, and BCR‐ABL1. *****Adjusted for for age at diagnosis, race, smoking status, Charlson, NPM1, FLT3, IDH, RUNX1, GATA2, BCR‐ABL1, ASXL1, TP53, and prognosis.

The analysis of treatment patterns (Table S2) showed that, compared to males, females were treated later (median days from diagnosis to first treatment of 18 [IQR 7–52] vs. 13 [4–36], *p* = 0.01), especially for chemotherapy (median days from diagnosis to first chemotherapy of 15.5 [IQR 6–37] vs. 13 [IQR 4–28], *p* = 0.03). Moreover, females were less likely to be prescribed decitabine (6% in females vs. 9.7% in males, *p* = 0.03) and had lower appointment completion rates (median of 68% [IQR 48–84] appointments attended for females vs. 75% [IQR 56–87] for males, *p* = 0.02). The likelihoods of receiving one of the types of treatment or being hospitalized (calculated via Cox proportional‐hazards regression, and Logistic Regression, respectively) were not different between the sexes in the AML population analyzed, including the sensitivity analyses by prognosis and year of diagnosis (Figure 1 and Table S3). However, in the subgroup of patients who had a record of hospitalization (*n* = 125), females were less likely to have a documentation of having received chemotherapy (females aHR = 0.61, 95% CI 0.40–0.93, Table S3).

### 3.3 Sex differences in treatment‐related adverse events

Among patients who received any kind of treatment, 36% of the females versus 30.9% of the males (*p* = 0.30) were diagnosed with a psychological affection (depression and/or anxiety and/or bipolar disorder), while 11.3% of the females versus 11.8% of the males (*p* = 0.99) had a diagnosis of cognitive decline/dementia after treatment (Table 2). For patients treated with chemotherapy (*n* = 406), 65.9% of the females versus 57.2% of the males (*p* = 0.09) had a documented chemotherapy adverse event, while those treated with immunotherapy had an irAEs diagnosed in 80% of the females versus 88.2% of the males (*p* = 0.98), and those treated with BMT had a complication recorded in 74.6% of the females versus 73.7% of the males (*p* = 1.00, Table 2). In patients with a record of hospitalization, females were 2.6 times more likely to have a chemotherapy adverse event (females aHR = 2.60, 95% CI 1.19–5.77, Figure 3).

**TABLE 2:** Sex differences in treatment adverse events, acute myeloid leukemia, University Hospitals (UH) population (2010–2022). A total of 1020 patients were analyzed with 42.6% females

	Male	Female	*p* value
	585 (57.4%)	435 (42.6%)	
Psychological affections, *n* (%)	76 (30.9%)	67 (36%)	0.30
Depression, *n* (%)	45 (18.3%)	39 (21%)	0.56
Anxiety, *n* (%)	49 (19.9%)	51 (27.4%)	0.08
Bipolar disorder, *n* (%)	2 (0.8%)	3 (1.6%)	0.75
Cognitive decline/dementia, *n* (%)	29 (11.8%)	21 (11.3%)	0.99
Chemotherapy, *n* (%)	236 (40.3%)	170 (39.1%)	—
Chemotherapy complications, *n* (%)	135 (57.2%)	112 (65.9%)	0.09
Adverse reaction, *n* (%)	37 (15.7%)	35 (20.6%)	0.25
Cardiomyopathy, *n* (%)	4 (1.7%)	2 (1.2%)	0.99
Diarrhea/enteritis, *n* (%)	38 (16.1%)	44 (25.9%)	0.02
Fatigue, *n* (%)	12 (5.1%)	12 (7.1%)	0.53
Nausea/vomiting, *n* (%)	19 (8.1%)	29 (17.1%)	0.008
Steatohepatitis, *n* (%)	7 (3%)	5 (2.9%)	1
Neuropathy, *n* (%)	10 (4.2%)	4 (2.4%)	0.45
Thrombocytopenia, *n* (%)	32 (13.6%)	19 (11.2%)	0.57
Lung disease, *n* (%)	28 (11.9%)	26 (15.3%)	0.39
Pain, *n* (%)	15 (6.4%)	14 (8.2%)	0.59
Anemia, *n* (%)	20 (8.5%)	24 (14.1%)	0.10
Agranulocytosis, *n* (%)	70 (29.7%)	59 (34.7%)	0.33
Mouth sore, *n* (%)	14 (5.9%)	22 (12.9%)	0.02
Dehydration/hypovolemia, *n* (%)	17 (7.2%)	19 (11.2%)	0.22
Renal failure, *n* (%)	2 (0.8%)	2 (1.2%)	1
Rash, *n* (%)	9 (3.8%)	14 (8.2%)	0.09
Infusion reaction, *n* (%)	16 (6.8%)	9 (5.3%)	0.68
Immunotherapy, *n* (%)	17 (2.9%)	10 (2.3%)	—
IRAES, *n* (%)	15 (88.2%)	8 (80%)	0.98
Hypothyroidism, *n* (%)	3 (17.6%)	3 (30%)	0.79
Hyperthyroidism, *n* (%)	0	1 (10%)	—
Hypophysitis/PGA, *n* (%)	0	0	—
Hyper/hypo‐ parathyroidism, *n* (%)	1 (5.9%)	0	—
AKI, *n* (%)	6 (35.3%)	4 (40%)	1
Neuritis, *n* (%)	5 (29.4%)	0	—
Hepatitis, *n* (%)	1 (5.9%)	0	—
Colitis, *n* (%)	0	1 (10%)	—
Pancreatitis, *n* (%)	0	0	—
Mucositis, *n* (%)	5 (29.4%)	3 (30%)	1
Arrhythmia, *n* (%)	5 (29.4%)	3 (30%)	1
Acute MI, *n* (%)	0	0	—
Myocarditis, *n* (%)	1 (5.9%)	0	—
Pericarditis, *n* (%)	0	0	—
Cardiomyopathy, *n* (%)	1 (5.9%)	0	—
Pneumonitis, *n* (%)	4 (23.5%)	4 (40%)	0.63
Type I diabetes, *n* (%)	0	1 (10%)	—
Meningitis, *n* (%)	0	0	—
Encephalitis, myelitis, encephalomyelitis, *n* (%)	0	0	—
Vitiligo, *n* (%)	0	0	—
BMT, *n* (%)	59 (10.1%)	57 (13.1%)	—
BMT complications, *n* (%)	44 (74.6%)	42 (73.7%)	1
Complications of BMT, NOS	14 (23.7%)	15 (26.3%)	0.91
BMT rejection	2 (3.4%)	1 (1.8%)	1
BMT failure	1 (1.7%)	0	—
BMT infection	0	0	—
BMT‐associated thrombotic microangiopathy	0	0	—
GVHD	43 (72.9%)	41 (71.9%)	1
Cytomegalovirus (CMV)	12 (20.3%)	12 (21.1%)	1
Hepatic veno‐occlusive disease (VOD)	0	0	—
BMT 30‐day mortality	1 (1.7%)	3 (5.3%)	0.58
BMT 60‐day mortality	3 (5.1%)	6 (10.5%)	0.45

*Note*: The *p*‐values are result of the Chi‐square test for categorical variables, and the Kruskal‐Wallis test for continuous variables.

Abbreviations: IQR, interquartile range; IRAES, Immune‐Related Adverse Events.

### 3.4 Sex differences in overall survival

**FIGURE 2:**
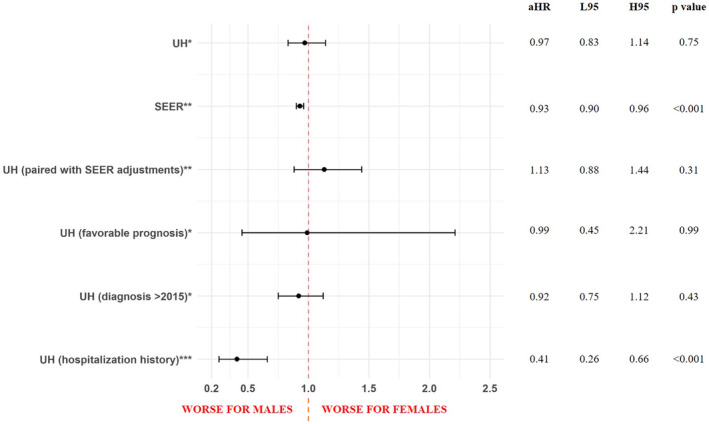
Forest plot representing sex differences in mortality, acute myeloid leukemia, University Hospitals (UH, 2010–2022) and SEER (2010–2019) populations. Results are presented in adjusted Hazard Ratios (aHR) for females, associated with 95% confidence intervals (L95, lower 95% confidence interval; H95, higher 95% confidence interval). *Adjusted for age at diagnosis, race, smoking status, Charlson score, TP53 mutation, prognosis, BMT, chemotherapy, psychological affections, and percentage of appointments attended. **Adjusted for age at diagnosis, race, ethnicity, time to treatment, and chemotherapy. ***Adjusted for age at diagnosis, race, smoking status, RUNX1, ASXL1, TP53, BMT, immunotherapy, number of admissions, BMT complications, IRAES, psychological affections, and percentage of appointments attended.

In this patient population overall, the median overall survival was 302 days [IQR 229–396] for females versus 325 days [IQR 275–416] for males (*p* = 0.78). No differences in the risk of death were observed (aHR for females = 0.96, 95% CI 0.82–1.12, Figure 2). The sensitivity analysis by prognosis and year of diagnosis showed similar results, while female patients with a record of hospitalization had a trend toward a longer median overall survival (2128 days [IQR 660‐NA] for females vs. 1102 days [IQR 611–3123] for males, *p* = 0.31) and lower risk of death (females aHR = 0.41, 95% CI 0.26–0.66, Table S4).

### 3.5 Predictors of sex differences in overall survival

**FIGURE 3:**
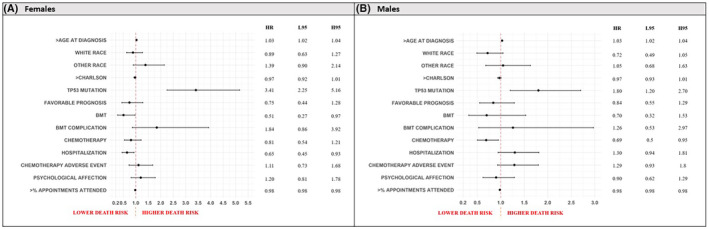
Forest plot representing predictors of all cause overall survival in females (A) and in males (B), acute myeloid leukemia, University Hospitals (UH) population (2010–2022). Results are presented in adjusted Hazard Ratios (aHR), associated with 95% confidence intervals (L95, lower 95% confidence interval; H95, higher 95% confidence interval).

Among all the patients analyzed, higher age at diagnosis (aHR = 1.03, 95% CI 1.02–1.04) and the presence of a TP53 mutation (aHR = 2.24, 95% CI 1.69–2.97) were associated with higher risks of death, while receipt of chemotherapy (aHR = 0.72, 95% CI 0.57–0.92), and higher appointment completion rates (aHR = 0.98, 95% CI 0.98–0.98) were associated with lower risk of death (Figure S3). Similar results were seen for males (Figure 3B).

Among females (Figure 3A), the variables associated with higher risk of death were similar to the general cohort, while BMT (aHR = 0.51, 95% CI 0.27–0.97), hospitalization record (aHR = 0.65, 95%CI 0.45–0.93), and higher appointment completion rates (aHR = 0.98, 95% CI 0.98–0.98) were associated with lower risk of death.

### 3.6 Predictors of sex differences in overall survival—patients with hospitalization record

**FIGURE 4:**
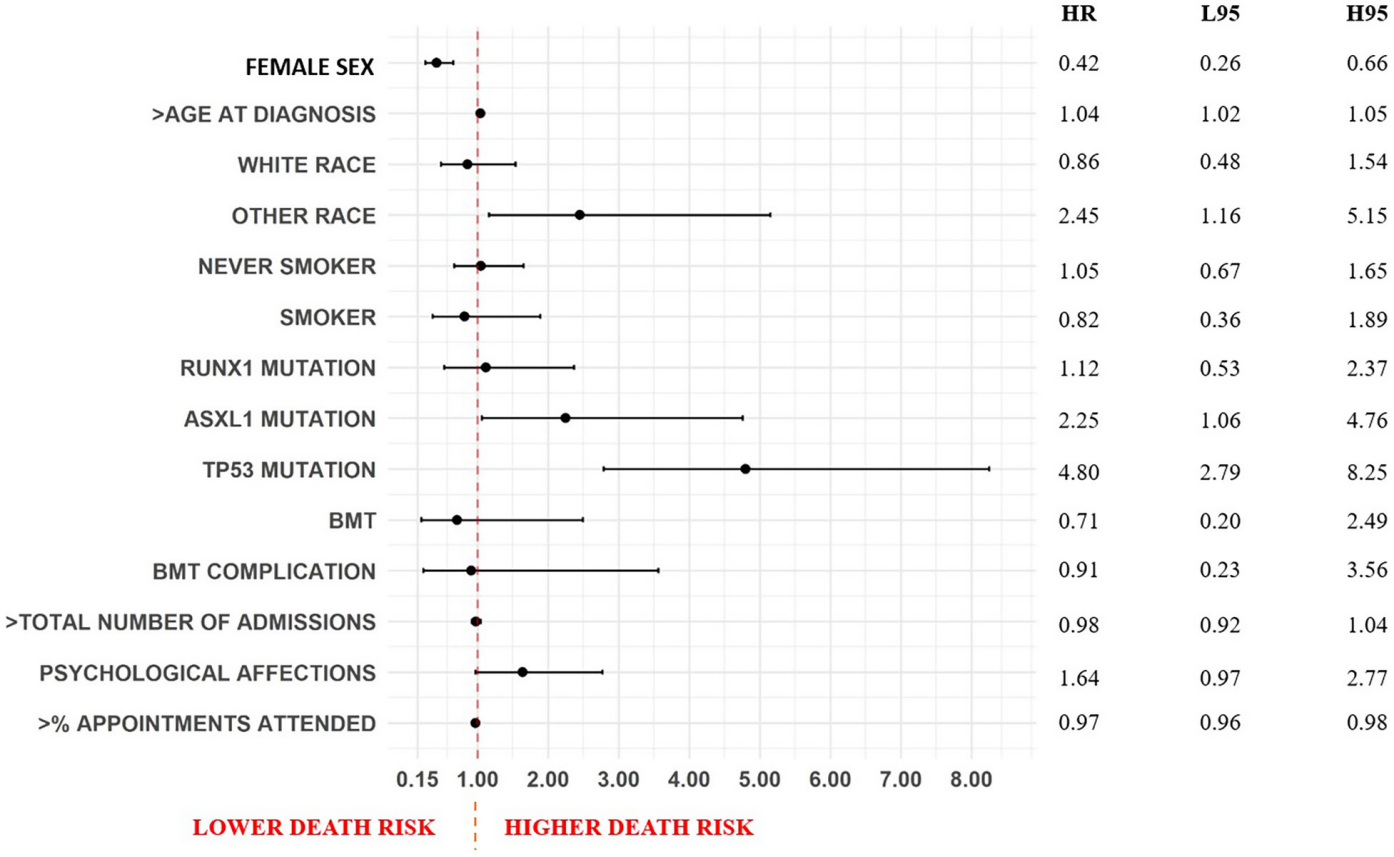
Forest plot representing predictors of overall survival in patients with hospitalization record, acute myeloid leukemia, University Hospitals (UH) population (2010–2022). Results are presented in adjusted Hazard Ratios (aHR), associated with 95% confidence intervals (L95, lower 95% confidence interval; H95, higher 95% confidence interval). BMT, bone marrow transplant.

In patients with at least one documented hospitalization (Figure 4), female sex (aHR = 0.42, 95% CI 0.26–0.66), and higher appointment completion rates (aHR = 0.97, 95% CI 0.96–0.98) were associated with lower risk of death, while higher age at diagnosis (aHR = 1.04, 95% CI 1.02–1.05), race other than Black or White (aHR = 2.45, 95% CI 1.16–5.15), ASXL1 mutation (aHR = 2.25, 95% CI 1.06–4.76), and TP53 mutation (aHR = 4.80, 95% CI 2.79–8.25) were associated with higher risk. In females, different from the general cohort, race was not associated with the outcome. Among males, ASLX1 mutation was not associated with higher risk of death, while White race was associated with lower risk (aHR = 0.35, 95% CI 0.15–0.77).

### 3.7 Comparison to the general US population

Using SEER data from 2010 to 2019 we analyzed 35,269 AML patients, of whom 44.6% (*n* = 15,719) were females (Table S5). The median age of this cohort was 70 (IQR 59–79) years, with a predominance of White (82.1%) and non‐Hispanic (88.2%) patients. The median time to treatment was 15 days (IQR 15–30), and 69.2% of the patients received chemotherapy. Females had lower risk of death (HR = 0.93, 95% CI 0.90–0.96, Figure 2).

## 4 DISCUSSION

The objective of this work was to carry out a comprehensive analysis of sex differences in AML in order to consolidate where these differences exist, and which factors play a relevant role in overall survival. We included 1020 patients (22,060 person‐months) over a 12‐year period and found a 1.34 male:female ratio, with a later start of treatment and lower treatment adherence rates in females, without differences in survival. Overall, age at diagnosis, race, TP53 mutation, chemotherapy, BMT, hospitalization, and compliance to treatment were associated with survival. Of these, BMT, hospitalization, and higher treatment completion rates were associated with lower risk of death in females.

In addition to our main findings, which remained similar in patients with favorable prognosis and with diagnosis after 2015, we also found that in patients with a hospitalization record, females were 39% less likely to receive chemotherapy, 160% more likely to have a chemotherapy adverse event, and had a 59% lower risk of death. In this cohort, race, ASXL1 mutation, TP53 mutation, and compliance to treatment were associated with survival.

The reasons for the sex differences in cancer incidence and survival are not yet well established and vary according to cancer type.^2^, ^7^, ^10^, ^26^, ^27^ It's reported that genetic polymorphisms (which is associated with drug metabolizing enzymes and the risk of carcinogenesis), epigenetics (marked by DNA methylation), hormones (especially estrogen levels), metabolism (related to nutrient utilization, and energy consumption and generation, involving glucose, amino acid, and reactive oxygen species [ROS]), TP53 (the most frequently mutated gene in cancer), cellular senescence, immunity, and angiogenesis are different between males and females and part of the explanation of the sex differences in cancer, highlighting a multifactorial role involved.^7^, ^27^, ^28^ Despite that, it's crucial to consider that, besides a lot of sex differences described in the literature, some mechanisms and pathways will not exhibit these differences and not all the differences seen will impact on cancer rates and survival.^27^

In HMs, there is an excess absolute rate (EAR) of 0.60 and poor survival outcomes in males.^7^, ^26^, ^27^ Males also have a higher AML incidence, which increases with age, while the survival data points to female advantage or equal risks between the sexes.^8^, ^12^, ^29^ Historically, the higher male incidence that increases with age was explained by occupational exposure, but this hypothesis is currently refuted, with considerations of multifactorial factors being the most likely contributors.^12^ In relation to survival, studies show that males with AML exhibit higher rates of RUNX1, ASXL1, SRSF2, BCOR, U2AF1, EZH2, and ZRSR2 mutations, while females have higher rates of FLT3‐ITD, NPM1, and DNMT3A, concluding that this sex have more high‐risk somatic mutations.^9^, ^30^

Our study supports prior findings about sex differences in AML incidence and mutations, corroborating with reports of higher incidence, and higher rates of RUNX1 and ASXL1 in males.^9^, ^12^ We also report a higher rate of smokers or former smokers, a known risk factor for AML, added to higher rates of people who are White, race known to have higher incidences of the disease, in the male population, and these variables may be part of the multiple factors involved in the differences in incidence.^31^, ^32^ In addition, the higher rate of patients who are Black females may be one of the reasons involved in the later treatment and lower compliance to treatment seen in the females from the general cohort, and in the lower chance of receiving chemotherapy and higher risk of chemotherapy adverse event in the females with records of hospitalization, since reports in the literature demonstrate that this race, due to social constructs, has worse indicators of social determinants of health (SDOH) and, therefore, experience worse health care and higher rates of adverse events.^33^, ^34^ It is also interesting to note that, although not statistically significant, our study reported higher rates of psychological affections in females, in agreement with the literature, a fact partially explained by better communication and connection of this sex with their providers.^14^, ^35^, ^36^

Our survival findings are also in agreement with the literature, following conflicting reports of female advantage and of equal risks between the sexes.^37^, ^38^ Interestingly, we reported both findings, with equal risks seen in the general population and female advantage in patients with hospitalization records and in the SEER data. Our analysis showed impact of age at diagnosis, race, mutations (especially TP53), and treatment patterns (including BMT, chemotherapy, hospitalization, and percentage of appointments attended) on sex differences in overall survival following AML diagnosis. These survival results allow us to hypothesize a female advantage, especially when there is a close follow‐up. The equal sex risks in survival seen in our general cohort may be explained by racial disparities, as evidence points that the Black race, especially due to the social construct, experiences worse outcomes.^39^

This study has several limitations. Some patients are referred to our institution for second opinions at the academic campus, after undergoing diagnosis and initial treatment, and our institutional database is EMR‐based, thus some of the information may be incomplete or inaccurate, and reflect bias due to the self‐reporting nature. Considering a single institution, some patients may have been to lost follow‐up or sought emergency care at other services. The 10‐year timeframe can encompass generational changes in cancer care. The use of ICD codes to define treatment adverse events can lead to underrepresentation. The available treatment variables did not allow us to stratify patients in relation to intensive chemotherapy. Some factors that may influence outcomes, such as alcohol drinking or addictive drugs use, were not available and could not be accounted. However, our database integrates multiple disparate sources, leading to rarely seen in other databases detailed and longitudinal information on each patient, and our institution, as an oncology center, maintain close patient's follow‐up, facts that, together with the robust statistical analyses, the sensitivity analysis in different groups, and the validation with a national database, counterbalance the limitations.

In summary, we found a higher incidence of AML in males, highlighted the impact of mutational factors, and demonstrated the importance of variables such as age, race, and treatment (including BMT, chemotherapy, hospitalizations, and compliance to treatment) in survival. In addition, we demonstrated female advantage in survival, especially when there is a close follow‐up. Future studies should focus on prospective designs, description of racial disparities, inclusion of different populations, and analysis of variables not yet described or analyzed in the literature in order to improve the understanding of the impact of each factor on the multifactorial hypothesis involved in the sex differences in AML.

## AUTHOR CONTRIBUTIONS

**Nickolas Stabellini:** Conceptualization (equal); data curation (lead); formal analysis (lead); methodology (equal); project administration (lead); writing – original draft (lead). **Ben Tomlinson:** Conceptualization (supporting); methodology (supporting); writing – review and editing (supporting). **Jennifer Cullen:** Methodology (equal); writing – review and editing (equal). **Alberto J Montero:** Conceptualization (equal); data curation (supporting); project administration (equal); resources (supporting); supervision (supporting); writing – review and editing (equal). **Kristin Waite:** Project administration (equal); writing – review and editing (equal). **John Shanahan:** Data curation (equal). **Jill S. Barnholtz‐Sloan:** Conceptualization (lead); data curation (equal); methodology (equal); project administration (equal); resources (equal); supervision (lead); writing – review and editing (equal). **Nelson Hamerschlak:** Conceptualization (equal); methodology (lead); project administration (equal); resources (equal); supervision (equal); writing – review and editing (equal).

## FUNDING INFORMATION

NS is supported through funding from the Sociedade Beneficente Israelita Brasileira Albert Einstein on the program "Marcos Lottenberg & Marcos Wolosker International Fellowship for Physicians Scientist ‐ Case Western." Jill S. Barnholtz‐Sloan is a full time, paid employee of the NCI/NIH. Kristin Waite is a full time, paid contractor of the NCI/NIH.

## CONFLICT OF INTEREST

None of the authors have any competing interests.

## ETHICAL APPROVAL INFORMATION

Patient records were deidentified, and the study was approved by the University Hospitals of Cleveland Institutional Review Board (IRB).

## Supporting information


Appendix S1

## Data Availability

University Hospitals (UH) Seidman Cancer Center database is available at University Hospitals Cleveland Medical Center and has access restricted to researchers with appropriate IRB approval.
