# Supplementary material for: Sex differences in adults with acute myeloid leukemia and the impact of sex on overall survival
Source: Cancer Med. 2022 Nov 23;12(6):6711–21. doi: 10.1002/cam4.5461 (PMC10067038; doi:10.1002/cam4.5461)
Supplement: Supplementary file 1 — Appendix S1 [file CAM4-12-6711-s001.docx]

**Title: Sex Differences in adults with acute myeloid leukemia and the impact of sex on overall survival.**

**Supplemental Material**

**Supplemental Figure 1.** Study consort diagram detailing inclusion and exclusion criteria for acute myeloid leukemia University Hospitals (UH) population (2010-2022). The final cohort included 1,020 patients, of which 435 were female.


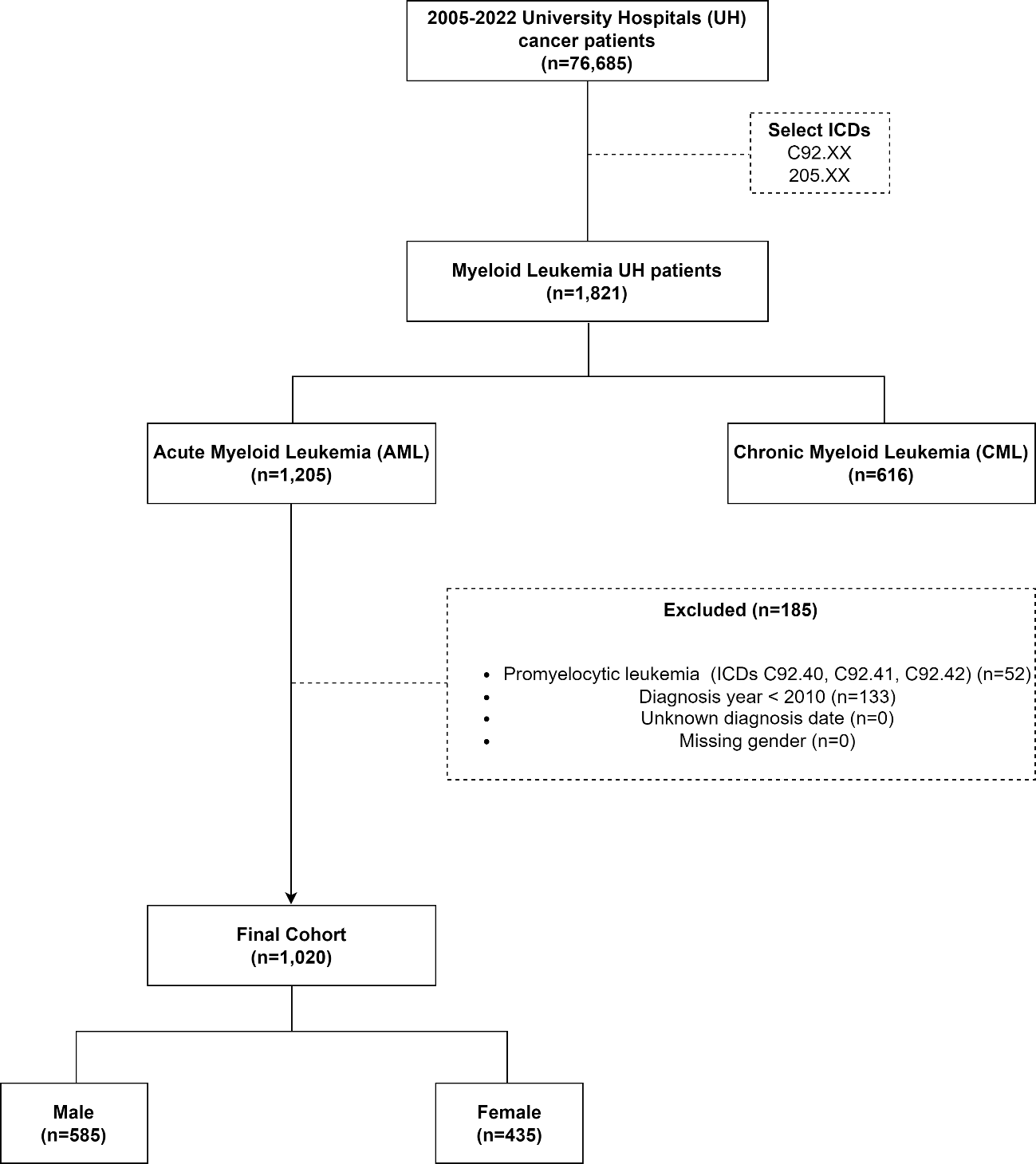


**Supplemental Figure 2.** Study design detailing multiple spectra of acute myeloid leukemia, University Hospitals (UH) population (2010-2022). Sex differences were studied for every spectrum and its impact in mortality accounted.


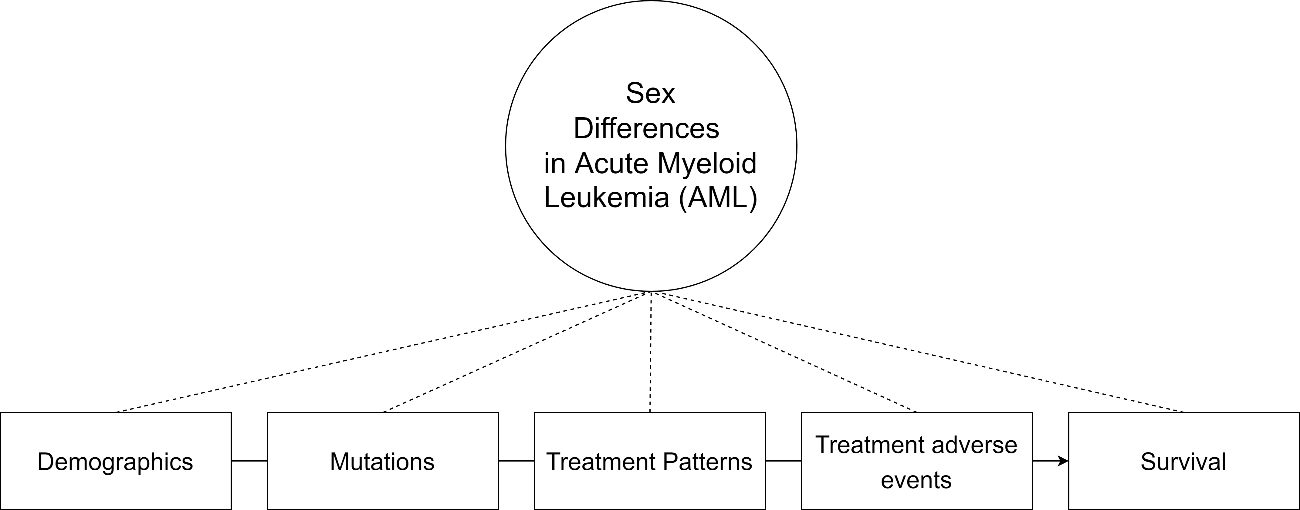


**Supplemental Figure 3.** Forest plot representing predictors of overall survival (all cause) , acute myeloid leukemia, University Hospitals (UH) population (2010-2022). Results are presented in adjusted Hazard Ratios (aHR), associated with 95% confidence intervals (L95=lower 95% confidence interval; H95=higher 95% confidence interval).


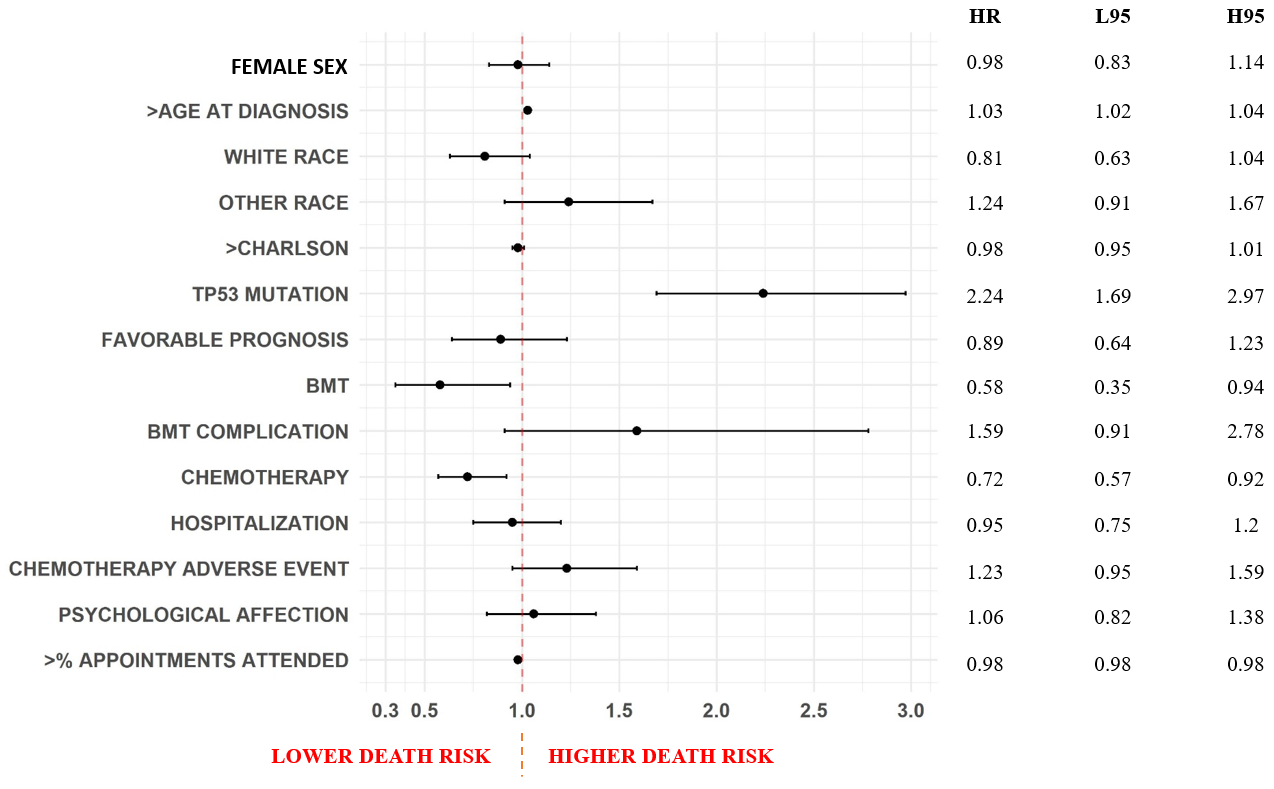


**Supplemental Table 1.** International Classification of Diseases (ICD) 9/10 codes used for categorization of chemotherapy adverse events, bone marrow transplant (BMT) complications, irAEs, psychological affections, and cognitive decline/dementia.

| **Chemotherapy** | |
| --- | --- |
| **Complication** | **ICD Codes** |
| Chemotherapy adverse reaction | T45.1, T88.7XXA; E933.1; 995.20; E947.9 |
| Chemotherapy-induced cardiomyopathy | I42.X; 425.4 |
| Chemotherapy-induced diarrhea/enteritis | R19.7; K52.1; K52.3; K52.8; K52.9; 558.9; 787.91 |
| Chemotherapy-induced fatigue | R53; 780.79 |
| Chemotherapy-induced nausea/vomiting | R11; 787.01; 536.2; 787.02; 787.03 |
| Chemotherapy-induced steatohepatitis | K75.81; K71.6; 571.8; 573.3 |
| Chemotherapy-induced neuropathy (and you may see peripheral neuropathy, sensor and motor neuropathy as well) | G62.X; G63.1; 357.7; 356.9; 355.9 |
| Chemotherapy-induced thrombocytopenia | D61.810; D69.59; 284.11; 287.49 |
| Chemotherapy-induced lung disease | J18.9, J84.89; 486; 516.8; 515 |
| Related Pain | G89.3; M25.50; 338.3; 338.29; 719.40 |
| Anemia due to chemotherapy | D64.81; D61.1; D63.0; 285.3; 284.89; 285.22 |
| Agranulocytosis secondary to cancer chemotherapy | D70. 1; D70.2; D70.8; D70.9; 288.50; 288.03; 288.00; 288.09 |
| Mouth sore secondary to chemotherapy | K12.30; K12. 31; K12.32; 528.00; 528.01; 528.02 |
| Dehydration/Hypovolemia | E86.X; 276.52; 276.51; 276.50 |
| Renal failure, drug induced | N14.1; N14.2; N14.4; 584.5 |
| Drug induced rash | L27.0, L27.1. L53.0, L27.8, L27.9, L56.0, L56.1; 693.0 |
| Infusion reaction | T80.X; V58.89; 999.9; 999.89; 999.88; 999.81; 999.80; 999.42; 999.33; 999.32; 999.31; 999.2; 909.3 |
| **IRAES** | |
| **Complication** | **ICD Codes** |
| Anemia | 285.3, 285.8, 285.9284.x, 283.x, D59.x, D61.x, D60.x, D64.2, D64.3, D64.8 |
| Thrombocytopenia | 287.3, 287.31, 287.8, 287.9, 287.31, 287.32, 287.4, 287.49, 287.5287.8, 287.9, D69.3, D69.41, D69.49, D69.59, D69.6 |
| Leukopenia | 288.00, 288.03, 288.09 288.4, 288.5x, 288.8, 288.9, D72.1, D72.81, D72.810, D72.818, D72.819, D70.9, D70.4, D70.2, D76.1, D76.3 |
| Hypothyroidism | 244.3, 244.8, 244.9, E03.2, E03.8, EO3.9 |
| Hyperthyroidism | 242.x, E05.x |
| Hypophysitis/PGA | 253.0, 253.1, 253.2, 253.4, 253.5, 253.6, 253.7, 253.8, 253.9, 255.0, 255.10, 255.11, 255.2, 255.3, 255.5, 255.41, 255.42, 255.5, 255.6, 255.8, 255.9, E22.x, E23.x, E27.x, E26.02, E26.09, E26.0, E26.1 E26.8, E26.89, E26.9, E24.x |
| Hyper/hypo- parathyroidism | 252.x, E21.x, E20.x |
| AKI | 584.x, 580.x, 581.x, 583.x N17.x, N00.x, N01.x, N04.x, N05.x N06.x, N14.1, N14.2 |
| Neuritis | 356.4, 356.8, 356.9, 357.4, 357.6, 357.7, 357.8, 357.9, 729.2, G60.3, G60.8, G60.9, G61.x, G62.0, G62.2, G62.9, G90.0, G90.0x, G90.2, G90.3, G90.4, M79.2 |
| Hepatitis | 573.3, 790.5, K71, R74.8, K75.4, K75.9, R94.5 |
| Colitis | 558.2, 558.3, 558.4, 558.9, 555, 556.8, 556.9, K52.1, 52.29, 52.3, 52.9, 52.89, 53.82 |
| Pancreatitis | 577.0, K85.x |
| Mucositis | 528.x, K12.30, K12.31 |
| Arrhythmia | 427.x I48.x, I47.x, I49.x, I46.x |
| Acute MI | 410.x, I21.x |
| Myocarditis | 422.x, 429.x, I40.x, I51.4, I51.8, I51. |
| Pericarditis | 420.x, 423.x, I30.x I31.4, I31.8-9 |
| Cardiomyopathy | 425.4, 425.9, I42.0, I42.7, I42.9 |
| Pneumonitis | 508.8, 508.9, 486.x, 516.3x, 516.9, J84.11x, J70.9, J70.8, J70.2, J70.3, J70.4, J18.9, J84.89, J84.9 |
| Type I diabetes | 250.0, 250.01, 250.03, 250.1, 250.11, 250.13, 250.2, 250.21, 250.23, 250.3, 250.31, 250.33, 250.4, 250.41, 250.43, 250.6, 250.61, 250.63, 250,7, 250.71, 250.73, 250.81, 250.83, 250.9, 250.91, 250.93, E09.x, E10.x, E13 |
| Meningitis | 047.9, 322.9, G03.9, A87.9 |
| Encephalitis, myelitis, encephalomyelitis | 323.8, 323.81, 323.82, 323.7, 323.71, 323.72, 323.9, G92, G04.81, G04.89, G04.90, G04.91 |
| Vitiligo | 709.00, 709.01, 709.09, L80, L81.8, L81.9, |
| Dysfunctional uterine bleeding and infertility | 626.0, 626.1, 626.2, 626.4, 626.5, 626.6, 626.7, 626.8, 626.9, 627.0, 627.1, 627.2, 627.4, 627.8, 627.9, 628.x N92.0, N92.1, N92.3, N92.4, N92.5, N92.6, N93.0, N93.9, N95.1, N95.8, N95.9, N97.x |
| **Psychological Affections** | |
| **Complication** | **ICD Codes** |
| Depression | F33, F32.89, F32.9, 311, 296.82, 296.33, 296.35, 296.30 |
| Anxiety | F41.9, F41.8, F41.1, 300.00, 300.4, 300.02 |
| Bipolar Disorder | F31, 296.80, 296.50, 296.89, 296.7 |
| **BMT** | |
| **Complication** | **ICD Codes** |
| Complications of BMT, NOS | T.86.0, T86.00, T86.09, T86.83, T86.838, T86.839, 996.85, 996.89 |
| BMT rejection | T86.01, T86.830 |
| BMT failure | T86.02, T86.831 |
| BMT infection | T86.03, T86.832 |
| BMT associated thrombotic microangiopathy | M31.11 |
| GVHD | D89.81X, 279.5X |
| Cytomegalovirus (CMV) | C25.X, 078.5 |
| Hepatic veno-occlusive disease (VOD) | K76.5, 573.8 |
| **Other Complications** | |
| **Complication** | **ICD Codes** |
| Cognitive Decline/Dementia | 331.83, 294.20, 290.10, 290.11, 290.13, 290.21, 290.8, 799.52, 780.93, 294.9, 799.5, G31.84, R41.X, F03.90 |

**Supplemental Table 2.** Sex differences in treatment patterns, acute myeloid leukemia, University Hospitals (UH) population (2010-2022). A total of 1,020 patients were analyzed, with 42.6% females. The p-values are result of Chi-square test for categorical variables, and Kruskal-Wallis test for continuous variables. IQR = interquartile range.

|  | **Male** | **Female** | **p value** |
| --- | --- | --- | --- |
|  | **585 (57.4%)** | **435 (42.6%)** |  |
| **Days from diagnosis to first treatment - median (IQR)** | 13 (4-36) | 18 (7-52) | 0.01 |
| **Days from diagnosis to chemotherapy - median (IQR)** | 13 (4-28) | 15.5 (6-37) | 0.03 |
| **Days from diagnosis to immunotherapy - median (IQR)** | 88 (26-136) | 80.5 (36-125) | 0.78 |
| **Days from diagnosis to bone marrow transplant - median (IQR)** | 90 (63-138) | 89 (61-138) | 0.84 |
| **Bone marrow transplant - n (%)** | 59 (10.1%) | 57 (13.1%) | 0.16 |
| Allogeneic - n (%) | 56 (9.6%) | 52 (12%) | 0.26 |
| Autologous - n (%) | 5 (0.9%) | 2 (0.5%) | 0.70 |
| **Chemotherapy - n (%)** | 236 (40.3%) | 170 (39.1%) | 0.73 |
| **Immunotherapy - n (%)** | 17 (2.9%) | 10 (2.3%) | 0.68 |
| **Cytarabine - n (%)** | 202 (34.5%) | 145 (33.3%) | 0.73 |
| **Daunorubicin - n (%)** | 31 (5.3%) | 22 (5.1%) | 0.97 |
| **Idarubicin - n (%)** | 79 (13.5%) | 49 (11.3%) | 0.33 |
| **Midostaurin - n (%)** | 18 (3.1%) | 12 (2.8%) | 0.91 |
| **Sorafenib - n (%)** | 6 (1%) | 8 (1.8%) | 0.40 |
| **Gemtuzumab ozogamicin - n (%)** | 11 (1.9%) | 7 (1.6%) | 0.93 |
| **Etoposide - n (%)** | 31 (5.3%) | 36 (8.3%) | 0.07 |
| **Fludarabine - n (%)** | 113 (19.3%) | 90 (20.7%) | 0.64 |
| **Clofarabine - n (%)** | 29 (5%) | 19 (4.4%) | 0.77 |
| **Mitoxantrone - n (%)** | 28 (4.8%) | 34 (7.8%) | 0.06 |
| **Venetoclax - n (%)** | 16 (2.7%) | 10 (2.3%) | 0.81 |
| **Azacitidine - n (%)** | 146 (25%) | 93 (21.4%) | 0.20 |
| **Decitabine - n (%)** | 57 (9.7%) | 26 (6%) | 0.03 |
| **Ivosidenib - n (%)** | 1 (0.2%) | 0 | - |
| **Enasidenib - n (%)** | 0 | 2 (0.5%) | - |
| **Hospitalization - n (%)** | 116 (19.8%) | 96 (22.1%) | 0.42 |
| Total admissions per patient - median (IQR) | 4 (2-6) | 4 (2-8) | 0.97 |
| Elective admissions per patient - median (IQR) | 1.5 (0-3) | 1 (0-3) | 0.52 |
| Non-elective admissions per patient - median (IQR) | 2 (1-4) | 2 (1-5) | 0.37 |
| Lenght of stay in days (LOS) per patient- median (IQR) | 8 (4-11) | 6 (3-10) | 0.14 |
| **% appointments attended - median (IQR)** | 75 (56,87) | 68 (46,84) | 0.02 |

**Supplemental Table 3.** Univariable and multivariable results for treatment patterns and treatment adverse events, acute myeloid leukemia, University Hospitals (UH) population (2010-2022). The p-values are result of Cox proportional-hazard models for all variables except hospitalization, which p-value is result from logistic regression. Results are presented in Hazard Ratios (HR) or Odds Ratios (OR), lowest 95% confidence interval (L95), highest 95% confidence interval (H95). BMT = bone marrow transplant; IRAES = Immune-Related Adverse Events.

|  | **Univariable** | | | | **Multivariable** | | | |
| --- | --- | --- | --- | --- | --- | --- | --- | --- |
|  | **HR** | **L95** | **H95** | **p value** | **HR** | **L95** | **H95** | **p value** |
| **BMT*** | 1.43 | 0.98 | 2.09 | 0.06 | 1.31 | 0.89 | 1.93 | 0.15 |
| **BMT complications*** | 0.98 | 0.64 | 1.52 | 0.95 | 1.15 | 0.71 | 1.88 | 0.55 |
| **Chemotherapy**** | 0.9 | 0.74 | 1.1 | 0.31 | 0.89 | 0.72 | 1.09 | 0.27 |
| **Chemotherapy complications**** | 1.43 | 1.03 | 1.97 | 0.02 | 1.33 | 0.96 | 1.85 | 0.08 |
| **Immunotherapy***** | 0.78 | 0.36 | 1.72 | 0.55 | 0.88 | 0.4 | 1.95 | 0.76 |
| **IRAES***** | 1.11 | 0.33 | 3.67 | 0.85 | 0.61 | 0.14 | 2.5 | 0.49 |
| **Psychological affections****** | 1.27 | 0.9 | 1.79 | 0.16 | 1.22 | 0.87 | 1.73 | 0.24 |
| **Cognitive decline/dementia****** | 0.91 | 0.51 | 1.62 | 0.76 | 0.82 | 0.45 | 1.49 | 0.52 |
|  | **OR** | **L95** | **H95** | **p value** | **OR** | **L95** | **H95** | **p value** |
| **Hospitalization******* | 1.14 | 0.84 | 1.55 | 0.38 | 1.23 | 0.87 | 1.73 | 0.23 |
| **FAVORABLE PROGNOSIS** | | | | | | | | |
|  |  | | | | **Multivariable** | | | |
|  |  |  |  |  | **HR** | **L95** | **H95** | **p value** |
| **BMT*** |  |  |  |  | 1.21 | 0.37 | 3.89 | 0.74 |
| **BMT complications*** |  |  |  |  | 1.58 | 0.35 | 7.05 | 0.54 |
| **Chemotherapy**** |  |  |  |  | 0.76 | 0.43 | 1.33 | 0.34 |
| **Chemotherapy complications**** |  |  |  |  | 2.74 | 0.93 | 8.09 | 0.06 |
| **Immunotherapy***** |  |  |  |  | 0.56 | 0.05 | 5.48 | 0.62 |
| **IRAES***** |  |  |  |  | - | - | - | - |
| **Psychological affections****** |  |  |  |  | 0.91 | 0.41 | 2.03 | 0.83 |
| **Cognitive decline/dementia****** |  |  |  |  | 0.75 | 0.13 | 4.09 | 0.74 |
|  |  |  |  |  | **OR** | **L95** | **H95** | **p value** |
| **Hospitalization******* |  |  |  |  | 0.37 | 0.12 | 1.06 | 0.07 |
| **HOSPITALIZATION HISTORY** | | | | | | | | |
|  |  | | | | **Multivariable** | | | |
|  |  |  |  |  | **HR** | **L95** | **H95** | **p value** |
| **BMT*** |  |  |  |  | 0.85 | 0.43 | 1.66 | 0.64 |
| **BMT complications*** |  |  |  |  | 1.37 | 0.60 | 3.12 | 0.44 |
| **Chemotherapy**** |  |  |  |  | 0.61 | 0.4 | 0.93 | 0.02 |
| **Chemotherapy complications**** |  |  |  |  | 2.6 | 1.19 | 5.77 | 0.01 |
| **Immunotherapy***** |  |  |  |  | 0.43 | 0.13 | 1.39 | 0.16 |
| **IRAES***** |  |  |  |  | 1.58 | 0.22 | 11.38 | 0.64 |
| **Psychological affections****** |  |  |  |  | 1.05 | 0.57 | 1.93 | 0.87 |
| **Cognitive decline/dementia****** |  |  |  |  | 0.61 | 0.22 | 1.69 | 0.34 |
|  |  |  |  |  | **OR** | **L95** | **H95** | **p value** |
| **Hospitalization******* |  |  |  |  | - | - | - | - |
| **DX>2015 (n=664)** | | | | | | | | |
|  |  | | | | **Multivariable** | | | |
|  |  |  |  |  | **HR** | **L95** | **H95** | **p value** |
| **BMT*** |  |  |  |  | 1.17 | 0.71 | 1.95 | 0.51 |
| **BMT complications*** |  |  |  |  | 1.41 | 0.71 | 2.81 | 0.31 |
| **Chemotherapy**** |  |  |  |  | 0.87 | 0.68 | 1.12 | 0.3 |
| **Chemotherapy complications**** |  |  |  |  | 1.01 | 0.64 | 1.61 | 0.95 |
| **Immunotherapy***** |  |  |  |  | 0.48 | 0.17 | 1.34 | 0.16 |
| **IRAES***** |  |  |  |  | 1.15 | 0.18 | 7.03 | 0.87 |
| **Psychological affections****** |  |  |  |  | 1.17 | 0.76 | 1.8 | 0.46 |
| **Cognitive decline/dementia****** |  |  |  |  | 1.11 | 0.59 | 2.07 | 0.73 |
|  |  |  |  |  | **OR** | **L95** | **H95** | **p value** |
| **Hospitalization******* |  |  |  |  | 1.09 | 0.74 | 1.6 | 0.63 |

*Adjusted for age at diagnosis, race, ethnicity, Charlson, FLT3 mutation, and prognosis.

**Adjusted for age at diagnosis, race, Charlson, and prognosis.

***Adjusted for race, FLT3, RUNX1.

****Adjusted for age at diagnosis, Charlson, and BCR-ABL1.

*****Adjusted for for age at diagnosis, race, smoking status, Charlson, NPM1, FLT3, IDH, RUNX1, GATA2, BCR-ABL1, ASXL1, TP53, and prognosis.

**Supplemental Table 4.** Univariable and multivariable results for overall survival, acute myeloid leukemia, University Hospitals (UH, 2010-2022) and SEER (2010-2019) populations. The p-values are result of Cox proportional-hazard models for all variables. Results are presented in Hazard Ratios (HR), lowest 95% confidence interval (L95), highest 95% confidence interval (H95).

|  | **Univariable** | | | | **Multivariable** | | | |
| --- | --- | --- | --- | --- | --- | --- | --- | --- |
|  | **HR** | **L95** | **H95** | **p value** | **HR** | **L95** | **H95** | **p value** |
| **UH*** | 0.97 | 0.83 | 1.14 | 0.77 | 0.97 | 0.83 | 1.14 | 0.75 |
| **SEER**** |  | | | | 0.93 | 0.90 | 0.96 | <0.001 |
| **UH (paired with SEER adjustments)**** |  |  |  |  | 1.13 | 0.88 | 1.44 | 0.31 |
| **UH (favorable prognosis)*** |  |  |  |  | 0.99 | 0.45 | 2.21 | 0.99 |
| **UH (diagnosis >2015)*** |  |  |  |  | 0.92 | 0.75 | 1.12 | 0.43 |
| **UH (hospitalization history)***** |  |  |  |  | 0.41 | 0.26 | 0.66 | <0.001 |
| *Adjusted for age at diagnosis, race, smoking status, Charlson score, TP53 mutation, prognosis, BMT, BMT complication, chemotherapy, psychological affections, and % appointments attended. | | | | | | | | |
| **Adjusted for age at diagnosis, race, ethnicity, time to treatment, and chemotherapy | | | | | | | | |
| *** Adjusted for age at diagnosis, race, smoking status, RUNX1, ASXL1, TP53, BMT, immunotherapy, number of admissions, BMT complications, IRAES, psychological affections, and % appointments attended. | | | | | | | | |

**Supplemental Table 5.** Sex differences acute myeloid leukemia, SEER population (2010-2010). A total of 35,269 patients were analyzed, with 44.6% females. The p-values are result of Chi-square test for categorical variables, and Kruskal-Wallis test for continuous variables. IQR = interquartile range.

|  | **Male** | **Female** |
| --- | --- | --- |
|  | **19,550 (55.4%)** | **15,719 (44.6%)** |
| **Age at diagnosis - median (IQR)** | 70 (60-78) | 69 (57-79) |
| **Race - n (%)** |  |  |
| Black | 1,482 (7.6%) | 1,460 (9.3%) |
| White | 16,279 (83.3%) | 12,682 (80.7%) |
| Other | 1,789 (9.2%) | 1,577 (10%) |
| **Ethnicity - n (%)** |  |  |
| Non-Hispanic | 17,364 (88.8%) | 13,756 (87.5%) |
| **Days from diagnosis to first treatment - median (IQR)** | 15 (15-30) | 15 (15-30) |
| **Chemotherapy - n (%)** | 13,645 (69.8%) | 10,744 (68.4%) |
